# REVIEWING DATA DISAGGREGATION IN AGING HEALTH RESEARCH: NIA GRANTS AND RECOMMENDATIONS FOR EQUITY

**DOI:** 10.1093/geroni/igae098.4277

**Published:** 2024-12-31

**Authors:** Moroni Fernandez Cajavilca, Matthew Lee, Lan Doan

**Affiliations:** New York University, New York, New York, United States; New York University Grossman School of Medicine, New York, New York, United States; NYU Langone Health, NYU Grossman School of Medicine, New York, United States

## Abstract

Current federal minimum standards for collecting and reporting race and ethnicity data often group diverse individuals into broad, monolithic categories. Despite advances in policy and practice and calls for inclusive research, data equity remains a significant issue in aging-related health research. Racially and ethnically diverse older adults are underrepresented in health research and rarely disaggregated by ethnicity in data collection, analysis, and reporting of aging-related health outcomes. Data disaggregation offers a promising approach to advancing data equity in aging research. We reviewed the National Institutes of Health Research Portfolio Online Reporting Tools Expenditures and Results (NIH RePORTER) database to identify extramural National Institute on Aging (NIA) grants from 1985 to 2024 that proposed disaggregating race and ethnicity data for aging research. We found only 12 NIH-funded awards, demonstrating the limited focus on this issue. Most grants that explicitly mentioned data disaggregation were awarded from 2015 onwards (n=11, 92%). Proposed data disaggregation by ethnicity in NIA-funded grants included Latino and Hispanic, Asian, Pacific Islander, and Arab ethnic groups. Grant funding mechanisms included investigator-initiated grants such as R01 grants (n=5, 42%) and predoctoral training grants/awards such as the F31 and R36 (n=3, 25%). Enhancing cultural congruence and methodological rigor in data collection and reporting that is reflective of the diversifying aging population is crucial for meaningfully addressing health equity. We provide recommendations to advance aging-related health research and highlight data disaggregation considerations for racially and ethnically minoritized communities that are historically under-engaged and underrepresented in aging health research.

